# Atherosclerotic Cardiovascular Disease Risk and Lipid-Lowering Therapy Requirement in China

**DOI:** 10.3389/fcvm.2022.839571

**Published:** 2022-03-28

**Authors:** Lei Bi, Jiayi Yi, Chaoqun Wu, Shuang Hu, Xingyi Zhang, Jiapeng Lu, Jiamin Liu, Haibo Zhang, Yang Yang, Jianlan Cui, Wei Xu, Lijuan Song, Yuanlin Guo, Xi Li, Xin Zheng

**Affiliations:** ^1^National Clinical Research Center for Cardiovascular Diseases, National Health Commission (NHC) Key Laboratory of Clinical Research for Cardiovascular Medications, State Key Laboratory of Cardiovascular Disease, Fuwai Hospital, Chinese Academy of Medical Sciences and Peking Union Medical College, National Center for Cardiovascular Diseases, Beijing, China; ^2^State Key Laboratory of Cardiovascular Disease, Fuwai Hospital, National Center for Cardiovascular Diseases, Chinese Academy of Medical Sciences, Peking Union Medical College, Beijing, China; ^3^Central China Sub-center of the National Center for Cardiovascular Diseases, Zhengzhou, China; ^4^National Clinical Research Center for Cardiovascular Diseases, Shenzhen, Coronary Artery Disease Center, Fuwai Hospital Chinese Academy of Medical Sciences, Shenzhen, China

**Keywords:** ASCVD risk, LDL-C goals, lipid-lowering therapy, simulation, statins, ezetimibe, PCSK9 inhibitor

## Abstract

**Background:**

Lipid-lowering therapy (LLT) is one of the key strategies for reducing the atherosclerotic cardiovascular disease (ASCVD) burden. However, little is known about the percentage of people in need of different LLT regimens to achieve optimal targets of low-density lipoprotein cholesterol (LDL-C), and the corresponding cost and benefit.

**Methods:**

We conducted a simulation study based on the data from the nationwide China PEACE MPP population cohort (2015–2020), from which we included 2,904,914 participants aged 35–75 years from all the 31 provinces in mainland China. Participants were grouped based on their 10-year ASCVD risks, then entered into a Monte Carlo model which was used to perform LLT intensification simulation scenarios to achieve corresponding LDL-C goals in each risk stratification.

**Results:**

After standardizing age and sex, the proportions of participants included at low, moderate, high, and very-high risk were 70.8%, 15.6%, 11.5%, and 2.1%, respectively. People who failed to achieve the corresponding LDL-C goals −8.1% at low risk, 19.6% at moderate risk, 53.2% at high risk, and 93.6% at very-high risk (either not achieving the goal or not receiving LLT)—would be in need of the LLT intensification simulation. After the use of atorvastatin 20 mg was simulated, over 99% of the population at low or moderate risk could achieve the LDL-C goals; while 11.3% at high and 24.5% at very-high risk would still require additional non-statin therapy. After the additional use of ezetimibe, there were still 4.8% at high risk and 11.3% at very-high risk in need of evolocumab; and 99% of these two groups could achieve the LDL-C goals after the use of evolocumab. Such LLT intensification with statin, ezetimibe, and evolocumab would annually cost $2.4 billion, $4.2 billion, and $24.5 billion, respectively, and prevent 264,170, 18,390, and 17,045 cardiovascular events, respectively.

**Conclusions:**

Moderate-intensity statin therapy is pivotal for the attainment of optimal LDL-C goals in China, and around 10–25% of high- or very-high-risk patients would require additional non-statin agents. There is an opportunity to reduce the rising ASCVD burden in China by optimizing LLT.

## Introduction

The burden of atherosclerotic cardiovascular disease (ASCVD) has increased rapidly and substantially in China, causing approximately 2.4 million deaths in 2016 (1), with one third attributed to high low-density lipoprotein cholesterol (LDL-C) (2). Solid evidence has supported the log-linear association between concentration of LDL-C and ASCVD risk (3). The current guidelines for the management of ASCVD (4, 5) and dyslipidemia (6, 7) recommend optimizing statin therapy to achieve LDL-C goals based on individuals' ASCVD risks, and suggest additional use of ezetimibe or proprotein convertase subtilisin/kexin type 9 (PCSK9) inhibitors to attain lower LDL-C levels in population at higher ASCVD risks. Although the situation of the suboptimal utilization of lipid-lowering therapy (LLT) and achievement of LDL-C goals in China have been well studied (8–11), little is known about the requirement of various LLT regimens for the targeted populations to achieve optimal LDL-C goals. Such knowledge of the treatment burden of LLT would be helpful for developing more precise population risk control strategies and healthcare resource allocation policies, which is essential for reducing the rising ASCVD burden in China.

Accordingly, we conducted a simulation study based on the data from the China Patient-Centered Evaluative Assessment of Cardiac Events Million Persons Project (China PEACE MPP), which was a national population-based screening project. Using an established simulation model which implemented stepwise LLT intensification algorithm, we sought to estimate the proportions of people requiring various LTT regimens for achieving optimal LDL-C goals based on their risk stratifications. We further investigated the relevant cost and benefit of the LLT intensification.

## Methods

### Study Design and Participants

The China PEACE MPP is a government-funded public health project designed to focus on cardiovascular disease risk in China, which has been described previously (12). Briefly, from September 2015 to March 2020, we selected 284 sites (168 rural counties, 116 urban districts) across all the 31 provinces in mainland China to reflect the diversity in geographic distribution, economic development, and population structure (see details in Supplementary Material). At each site, participants aged 35–75 years who had lived in the region for at least 6 of the preceding 12 months were encouraged to participate in the study by local staff via extensive publicity campaigns. The overall response rate was around 30%. In these participants, the proportions of female and elderly were larger, compared with that in the entire national population aged between 35 and 75 years (Supplementary Figure 1).

**Figure 1:**
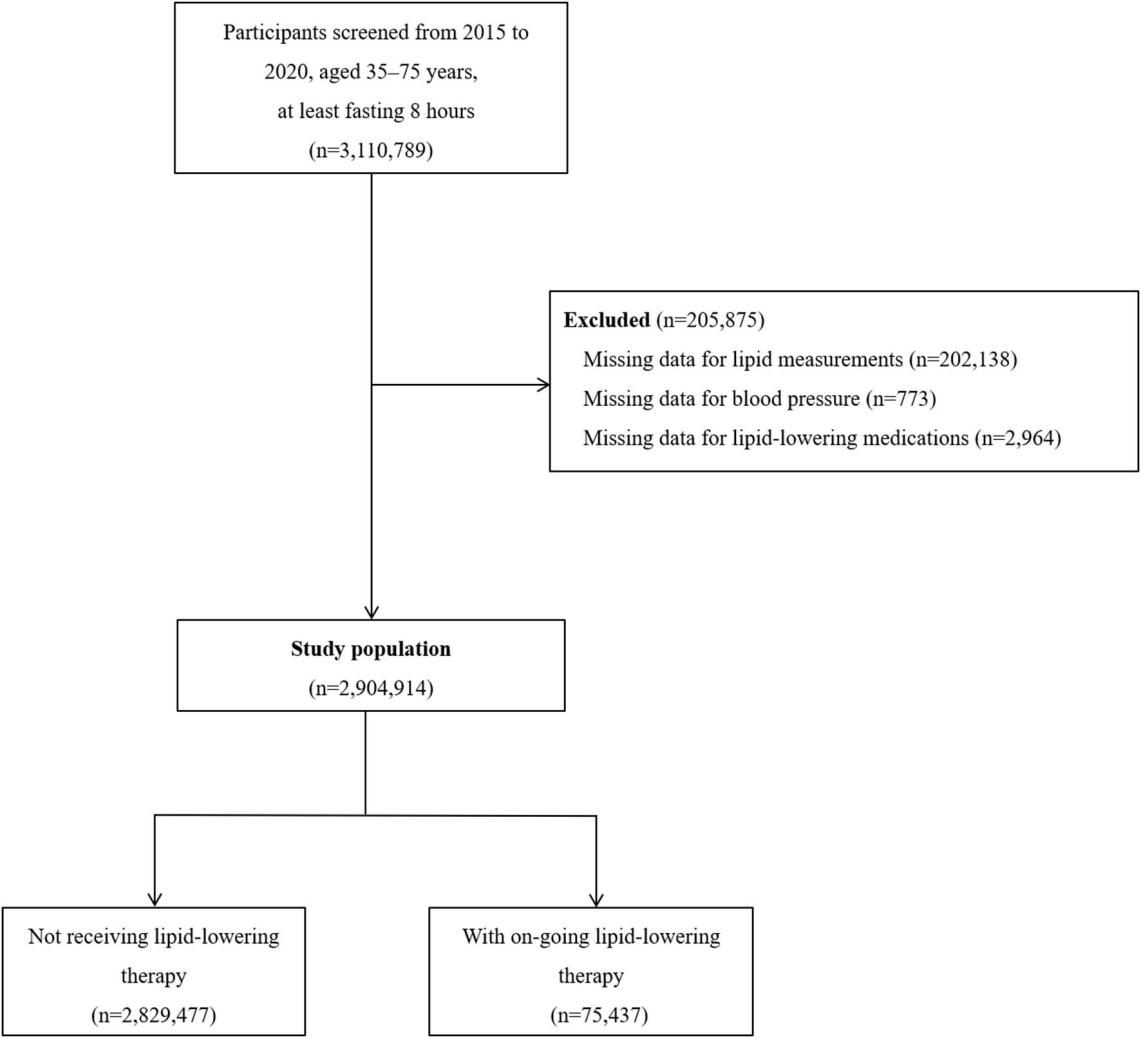
Flowchart for the study population.

Of the 3,110,789 participants who were fasting for at least 8 h, we excluded 205,875 (6.6%) who had missing data for blood lipid measurement, blood pressure measurement, or lipid-lowering medications use (Figure 1). The characteristics of the participants excluded are shown in Supplementary Table 1. The study complies with the principles that conveyed in the Declaration of Helsinki. The central ethics committee at the Fuwai Hospital approved this project and all enrolled participants provided written informed consent.

### Data Collection and Variables

At the initial screening visit of China PEACE MPP, each participant received a blood lipid test performed by a rapid lipid analyzer using the whole blood samples (CardioChek PA Analyzer; Polymer Technology Systems, Indianapolis, Indiana, USA). Total cholesterol (TC), triglycerides (TG), and high-density lipoprotein cholesterol (HDL-C) were measured, based on which LDL-C was calculated using the Friedewald equation (13). During the in-person interviews, information on socio-demographic characteristics, lifestyles, medical history, and medication history were collected by trained personnel. For each self-reported lipid-lowering medication, we collected information about its generic name, brand name, and daily dosage, then further confirmed that by requiring the participants to bring their drug packaging (boxes) to the baseline interview clinics.

Participants in the current study were grouped based on their ASCVD risks which was estimated using the 10-year ASCVD risk estimation chart recommended in the 2016 Chinese Guideline for the Management of Dyslipidemia in Adults (7) (Supplementary Figure 2). The LDL-C goals were recommended as an LDL-C level of <1.8 mmol/L for very-high-risk patients, <2.6 mmol/L for high-risk patients, and <3.4 mmol/L for low- or moderate-risk individuals. For LLT in Chinese population, the guideline (7) and expert consensus (14) for the management of dyslipidemia recommend an initial use of moderate-intensity statin and add-on therapy with ezetimibe and PCSK9 inhibitor for patients at high or very-high risk who are not achieving the LDL-C goals.

### Simulation of Lipid-Lowering Therapy

We used a Monte Carlo simulation model, a method for estimating outcomes through repeated random samplings, to simulate LLT intensification scenarios to achieve various LDL-C goals according to the participant's ASCVD risk. The model was based on the methods and data which have been previously presented and validated by Cannon et al. (15), and was conducted with the assumption of participants having received sufficient lifestyle modification, without intolerance issues and with full adherence. Given that there were participants with self-reported LLT but still eligible for the LLT simulation (i.e., not achieving the LDL-C goals), we assumed those not taking guideline-recommended lipid-lowering medications (i.e., statin, Xuezhikang, or ezetimibe), or not recalling the names of the medications, as not receiving LLT. The characteristics of the participants with self-reported LLT are shown in Supplementary Table 2.

In the base-case scenario, we simulated an initial use of atorvastatin 20 mg, followed by an additional use of ezetimibe 10 mg, and then evolocumab 140 mg biweekly if needed. We did not uptitrate the dose of statin, since the Chinese guidelines (7) noted that high-intensity statins should only be used with caution, as Chinese population are less tolerant to high-intensity statins. The logic of the base-case scenario is shown in Supplementary Figure 3. The achieved level of LDL-C was modeled probabilistically from the distribution of LDL-C level reduction with a given LLT. The effect of lipid-lowering medications used in the simulation was sampled from β probability density functions (Supplementary Table 3) derived from clinical trials, and was specific for each drug and dose. Thus, each participant followed a unique path in the simulation model, depending on their LDL-C levels at baseline and the probabilistic sampling of LDL-C level reduction.

We also performed additional analyses that represented scenarios with various assumptions based on the recommendations in the guideline (7) and expert consensus (14). In scenario S1, the threshold of initiating statins was LDL-C ≥ 4.1 mmol/L for participants at low or moderate risk. In scenario S2, if the LDL-C goals were not achieved with add-on ezetimibe, then evolocumab was used without ezetimibe. In scenario S3, for patients at very-high risk with persistently elevated LDL-C levels after statin and ezetimibe therapy, we replaced the threshold for intensification to evolocumab by a 50% reduction relative to the baseline LDL-C level before any LLT. In scenario S4, for patients at very-high risk who had a level of LDL-C <1.8 mmol/L before using LLT, the LDL-C goal was an additional 30% reduction relative to the LDL-C level at baseline. In scenario S5, the patients who did not achieve the LDL-C goals after the use of atorvastatin 20 mg would undergo uptitration to atorvastatin 40–80 mg before augmenting with ezetimibe and evolocumab. In scenario S6, we used alirocumab 75 mg biweekly instead of evolocuamb at the last step in the LLT intensification. In scenario S7, we set an LDL-C goal of <1.4 mmol/L and an LDL-C reduction of ≥50% from baseline for patients at very-high risk.

### Statistical Analyses

First, we estimated the proportion of participants at different ASCVD risks, and described the characteristics as means (standard deviation [SD]) and proportions as appropriate. Next, we estimated the proportion of LLT use, the distribution of LDL-C levels, and the proportions of participants achieving the LDL-C goals before and after the full LLT intensification simulation. We performed the simulation 1,000 times, then presented the mean values as the point estimates of the proportions of receiving LLT and achieving the LDL-C goals at each step, and reported the 95% confidence interval (CI) based on the distributions. Finally, we standardized these results according to the 2010 population census of China (16), and assessed the population-based cost and benefit of different LLT regimens.

The prices of statins were derived from the latest national centralized medicine procurement policy bid-winning announcement file (17). China pursued this policy in 2019 to lower medicine prices through competitive bidding, bulk purchasing, and reduced transaction costs. Ezetimibe and evolocumab have not been included in the policy, thus, their prices were derived from the lowest winning bid prices in the local government procurement catalogs (18). The average cost was $0.07 for atorvastatin 20 mg and $1.04 for ezetimibe 10 mg per day, and $198.8 for evolocumab 140 mg biweekly (1$ = 6.53 Chinese Yuan). The specific 10-year risk of ASCVD among participants without established ASCVD was calculated using the Chinese 10-year ASCVD risk assessment equation (19); and the incidences of recurrent cardiovascular events (CVE) among very-high-risk patients were derived from the result in previous study (20). We assumed a constant 22% relative risk/event reduction per 1 mmol/L reduction in LDL-C (3, 21), calculated as [1–0.78^(absolute reduction in LDL−C in mmol/L)^]. And the number of prevented events was calculated by summing the absolute risk differences between pre- and post-treatments among the participants receiving their corresponding LLT.

Analyses of the participant characteristics were performed with SAS 9.4, and the simulation was performed with R 4.0 and Python 3.8.

## Results

**Figure 2:**
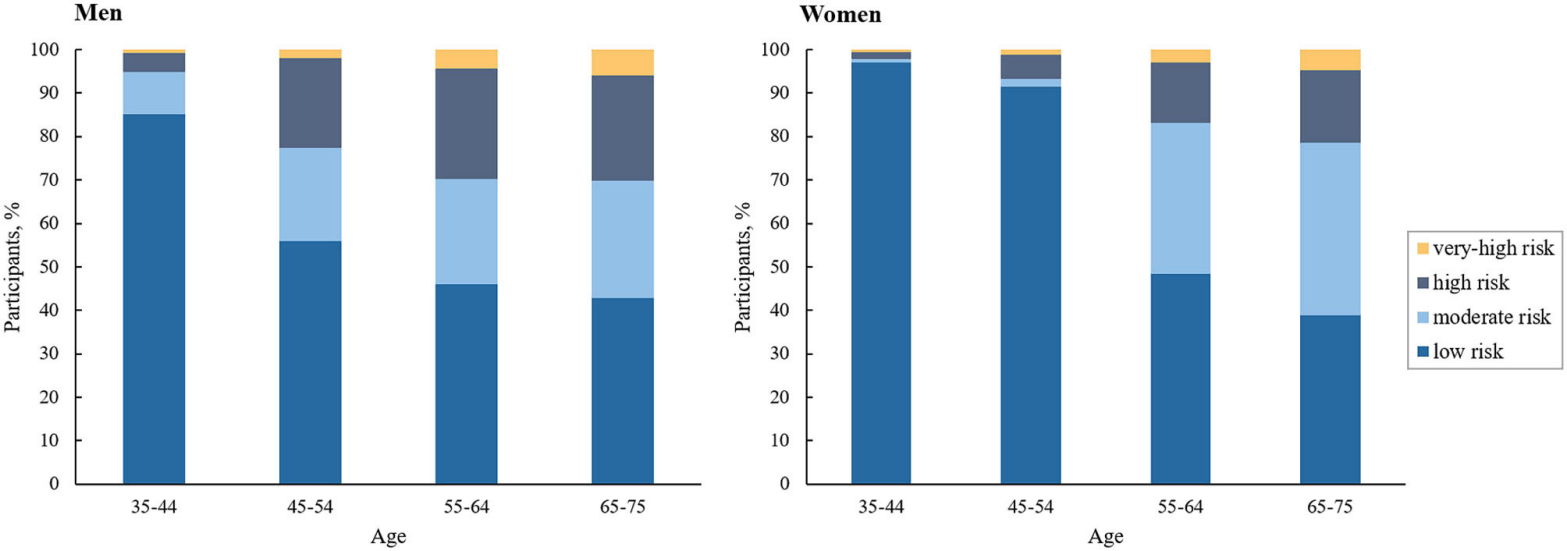
Proportion of participants at different 10-year ASCVD risks.

Our study sample contained 2,904,914 participants (60.6% women; mean [SD] age, 55.8 [9.9] years). About 62.2% (1,806,375) of the overall participants were at low ASCVD risk, 20.8% (603,351) moderate risk, 14.2% (412,961) high risk, and 2.8% (82,227) very-high risk. Table 1 provides a summary of the characteristics of the participants at diverse ASCVD risks. ASCVD risk increased with age in both men and women, and men had a higher proportion at high or very-high risk compared with women across all age groups (Figure 2). At baseline, the proportions achieving the corresponding LDL-C goals were 91.9, 80.4, 46.8, and 31.8% in participants at low, moderate, high, and very-high risk, respectively. Among those failing to achieve the LDL-C goals, the mean (SD) LDL-C levels were 3.83 (0.34) mmol/L, 3.84 (0.35) mmol/L, 3.59 (0.93) mmol/L, and 2.76 (0.74) mmol/L, respectively; and the proportions of LLT use were only 1.1, 2.0, 4.3, and 14.4%, respectively (Supplementary Table 4).

**Table 1:** Characteristics of the study population by 10-year ASCVD risk stratifications.

**Characteristics**	**Overall**	**Low risk**	**Moderate risk**	**High risk**	**Very-high risk**	***P* for trend**
Participants, N (%)	2,904,914 (100)	1,806,375 (62.2)	603,351 (20.8)	412,961 (14.2)	82,227 (2.8)	
Age group, years						<0.0001
35–44	431,107 (14.8)	398,513 (22.1)	18,095 (3.0)	12,060 (2.9)	2,439 (3.0)	
45–54	900,223 (31.0)	706,162 (39.1)	80,284 (13.3)	100,566 (24.4)	13,211 (16.1)	
55–64	913,671 (31.5)	433,690 (24.0)	279,956 (46.4)	168,273 (40.7)	31,752 (38.6)	
65–75	659,913 (22.7)	268,010 (14.8)	225,016 (37.3)	132,062 (32.0)	34,825 (42.3)	
Women	1,760,250 (60.6)	1,191,270 (66.0)	351,573 (58.3)	176,173 (42.7)	41,234 (50.2)	<0.0001
Urbanity						0.6720
Urban	1,160,575 (40.0)	728,781 (40.3)	228,166 (37.8)	169,766 (41.1)	33,862 (41.2)	
Rural	1,744,339 (60.0)	1,077,594 (59.7)	375,185 (62.2)	243,195 (58.9)	48,365 (58.8)	
Region						<0.0001
Eastern	1,211,146 (41.7)	718,415 (39.8)	261,891 (43.4)	193,585 (46.9)	37,255 (45.3)	
Central	678,563 (23.4)	408,045 (22.6)	153,031 (25.4)	97,432 (23.6)	20,055 (24.4)	
Western	1,015,205 (34.9)	679,915 (37.6)	188,429 (31.2)	121,944 (29.5)	24,917 (30.3)	
Household income, Yuan/year						<0.0001
<10,000	540,657 (18.6)	316,832 (17.5)	130,841 (21.7)	75,267 (18.2)	17,717 (21.6)	
10,000–50,000	1,580,899 (54.4)	989,607 (54.8)	322,782 (53.5)	223,108 (54.0)	45,402 (55.2)	
>50,000	502,332 (17.3)	322,879 (17.9)	91,448 (15.2)	74,838 (18.1)	13,167 (16.0)	
Unknown^*^	281,026 (9.7)	177,057 (9.8)	58,280 (9.6)	39,748 (9.7)	5,941 (7.2)	
Health insurance status						<0.0001
Insured	2,839,141 (97.7)	1,762,016 (97.5)	591,243 (98.0)	404,670 (98.0)	81,212 (98.8)	
Uninsured	17,786 (0.6)	12,158 (0.7)	3,040 (0.5)	2,311 (0.6)	277 (0.3)	
Unknown^*^	47,987 (1.7)	32,201 (1.8)	9,068 (1.5)	5,980 (1.4)	738 (0.9)	
Lipid levels, mmol/L						
Triglycerides (IQR)	1.33 (0.92)	1.23 (0.79)	1.46 (1.00)	1.64 (1.23)	1.42 (0.96)	<0.0001
Total cholesterol (SD)	4.56 (1.03)	4.32 (0.92)	4.95 (0.86)	5.12 (1.31)	4.41 (1.09)	<0.0001
HDL cholesterol (SD)	1.43 (0.40)	1.45 (0.39)	1.46 (0.40)	1.36 (0.43)	1.35 (0.38)	<0.0001
LDL cholesterol (SD)	2.42 (0.87)	2.24 (0.78)	2.71 (0.77)	2.84 (1.09)	2.31 (0.92)	<0.0001
Lipid-lowering therapy^†^	75,437 (2.6)	23,116 (1.3)	14,104 (2.3)	22,305 (5.4)	15,912 (19.4)	<0.0001
Cardiovascular risk factors						
Hypertension	1,368,604 (47.1)	420,946 (23.3)	549,757 (91.1)	336,938 (81.6)	60,963 (74.1)	<0.0001
Diabetes mellitus	219,067 (7.5)	13,109 (0.7)	244 (0.04)	190,076 (46.0)	15,638 (19.0)	<0.0001
Current smoker	565,515 (19.5)	248,258 (13.7)	113,614 (18.8)	185,343 (44.9)	18,300 (22.3)	<0.0001
Obesity^‡^	482,507 (16.6)	243,000 (13.5)	126,515 (21.0)	94,501 (22.9)	18,491 (22.5)	<0.0001

*Data are N (%) if not otherwise indicated. Triglycerides are shown as median (IQR), and other lipid levels are shown as means (SD)*.

* *Participants either refused to answer the question or did not know the answer*.

† *Defined as self-reported on-going lipid-lowering therapy*.

‡ *Defined as Body mass index ≥28 kg/m^2^*.

*ASCVD, atherosclerotic cardiovascular disease; HDL, high-density lipoprotein; LDL, low-density lipoprotein; IQR, interquartile range; SD, standard deviation*.

### Simulation of Lipid-Lowering Therapy

**Figure 3:**
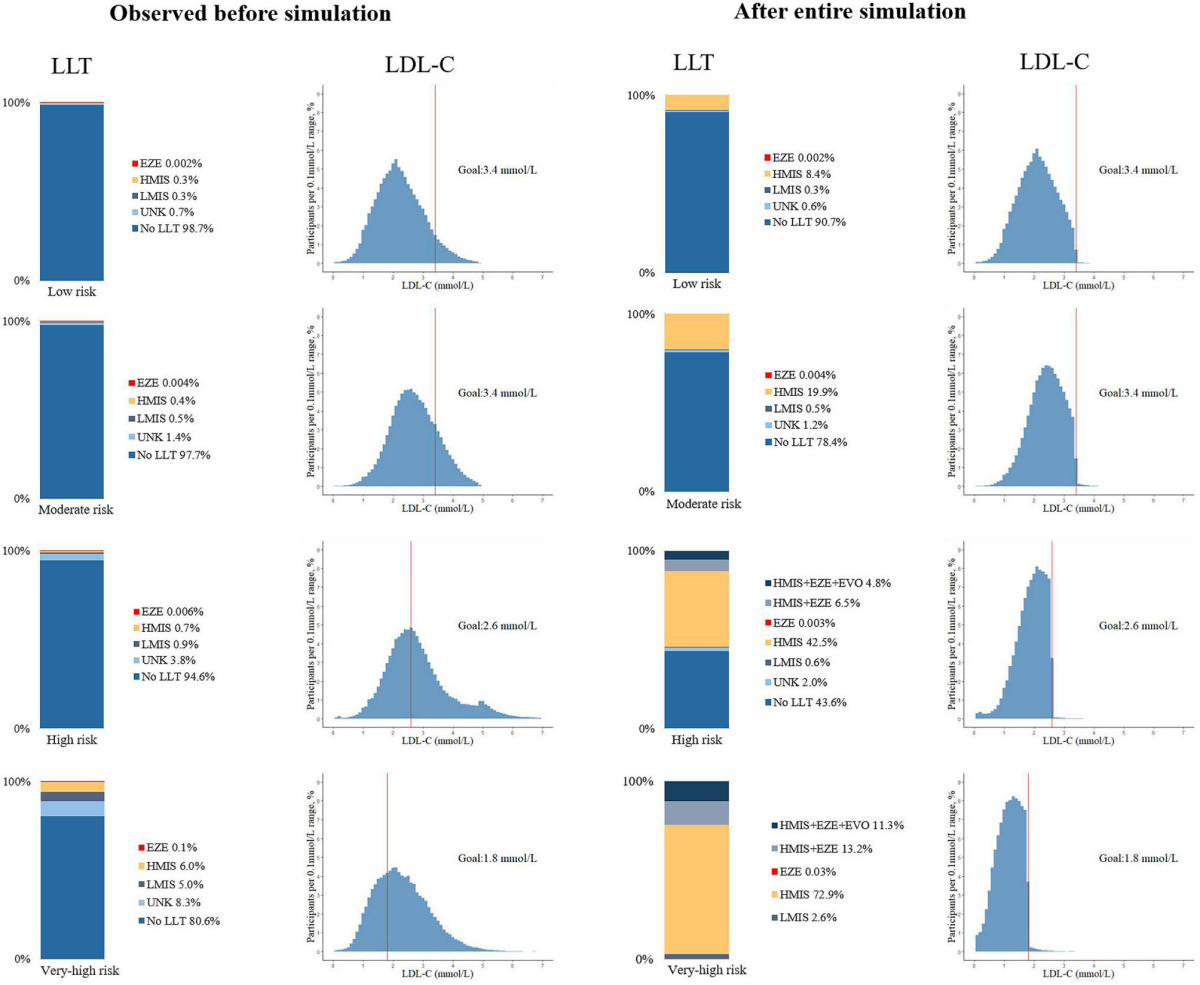
Use of lipid-lowering medications and distribution of LDL-C levels before and after the lipid-lowering therapy simulation, by 10-year ASCVD risk stratifications. UNK, unknown name or not guideline-recommended medications; HMIS, maximized uptake of moderate-intensity statins, including: atorvastatin 20 mg, simvastatin 40 mg, rosuvastatin 10 mg, pravastatin 40 mg, pitavastatin 4 mg, lovastatin 40 mg, or fluvastatin 80 mg; LMIS, statins with doses less than HMIS; EZE, ezetimibe; EVO, evolocumab 140 mg, biweekly.

Figure 3 shows the proportion of LLT use and the distribution of LDL-C levels in the participants at diverse ASCVD risks before and after the full LLT intensification in the base-case scenario. Changes in these measures at each step are shown in Supplementary Figure 4.

When the use or uptitration of atorvastatin 20 mg was simulated, over 99% of all the participants at low and moderate risk could achieve the LDL-C goals; while 11.3% at high risk and 24.5% at very-high risk would be in need of additional non-statin therapy, as they failed to achieve the corresponding LDL-C goals. After adding ezetimibe in these eligible participants, there remained 4.8% of participant at high risk and 11.3% at very-high risk in need of additional evolocumab. Then, after the use of evolocumab, over 99% in both groups could achieve the LDL-C goals. In total, 45.6% of the overall high-risk patients did not require the LLT; 43.1% required statin monotherapy; 6.5% statin and ezetimibe; and 4.8% add-on evolocumab. And 75.5% of the overall very-high risk patients required statin monotherapy; 13.2% statin and ezetimibe; and 11.3% add-on evolocumab. The 95% CIs based on the proportion of LLT use and LDL-C goal achievement at each step of the intensification are shown in Supplementary Tables 5, 6.

The corresponding results for men and women in the base-case scenario are shown in Supplementary Tables 7–9. The mean levels of LDL-C at each step in the base-case scenario and scenarios S1to S7 are shown in Supplementary Table 10; and the proportions of LLT use and LDL-C goal achievement in scenarios S1 to S7 are shown in Supplementary Tables 11, 12.

### Estimation of Population-Based Cost and Benefit

After standardizing age and sex using the data in the 2010 population census of China, it was estimated that among the 647 million Chinese population aged 35–75 years, the numbers of people at low, moderate, high, and very-high risk were estimated to be 458 million (70.8%), 101 million (15.6%), 74.4 million (11.5%), and 13.6 million (2.1%), respectively. The total number in need of LLT was 109 million (16.9%), including 12.3 million who were already taking LLT. And the numbers of annual new ASCVD cases in eligible individuals at low, moderate, and high risk before the LLT simulation were estimated to be 107,000, 157,000, and 472,000 respectively; and the recurrent CVE in eligible patients at very-high risk were 218,000.

According to the standardized result of the simulation, 101 million people would require maximized uptake of moderate-intensity statins, in whom 11 million would require additional ezetimibe, and 4.7 million add-on PCSK9 inhibitor. LLT intensification with these three medications would annually cost $2.4 billion, $4.2 billion, and $24.5 billion, respectively; and prevent 264,170, 18,390, and 17,045 CVE, respectively.

## Discussion

In this population-based mega screening project, we found around one seventh of Chinese adults aged 35–75 years were at high or very-high ASCVD risk; and one sixth were in need of LLT. As the first study to estimate the proportion of people requiring different LLT regimens for reducing ASCVD risk in China, we found most people could achieve LDL-C goals with maximized uptake of moderate-intensity statins, which could annually prevent 0.26 million CVE in nearly 100 million Chinese population at a relatively low cost.

Based on the large sample size and geographic coverage, our study provided population-based panorama of ASCVD risk stratifications and LLT gaps. We found that the proportion of high-risk population in China was much lower than that in Western countries. One study in the US reported that the proportion of individuals with 10-year risk ≥7.5% was around 40% (22). In contrast, the prevalence of ischemia heart disease or ischemia stroke (i.e., very-high-risk patients in our study) in China was higher than that in the US (23), indicating the poor risk mitigation in high-risk population. Moreover, in line with prior study (24), we found that despite over half of high-risk participants had LDL-C uncontrolled, only about 5% of them were receiving LLT. These findings highlighted an opportunity to improve primary prevention for ASCVD by optimizing LLT in China.

Comparing with two prior simulation studies in Western populations, which reported that 30–50% of patients with ASCVD required non-statin LLT in addition to atorvastatin 80 mg (15, 25), the proportion who were in need of non-statin therapy in our study was lower. The possible reason is that Chinese population, both with or without established ASCVD, has lower LDL-C levels than Western population (26). Nevertheless, the age-standardized incidence and mortality of ischemia heart disease or ischemia stroke were higher in China (23), suggesting that for a given LDL-C level, Chinese may have a higher risk of ASCVD in comparison with Western populations (26). Although this might be largely due to the suboptimal management of other risk factors (e.g., tobacco use, blood pressure, etc.), further studies are needed to explore whether lower LDL-C thresholds for initiating LLT as well as lower achievement goals are appropriate to reduce the risk of ASCVD in Chinese population.

Based on the simulation, our study further investigated the relevant population-based cost and benefit of the LLT intensification. We estimated that the cost of one prevented event would be $9,140 for atorvastatin, $227,230 for ezetimibe, and $1,435,550 for evolocumab, reflecting the relative cost-effectiveness of statin therapy. Pandya et al. (27) also showed that initiating statin therapy at lower risk thresholds remained cost-effective. Meanwhile, our findings demonstrated that LLT intensification could result in a 3% population-based absolute risk reduction for cardiovascular events over 10 years, highlighting the crucial role of sufficient LLT in achieving China's national plan for cardiovascular disease control which aims at reducing the age-standardized mortality rate of cardiovascular disease by 15% compared with 2015 by 2025 (28).

Our results have substantial implications for policies. First, making the best use of affordable essential drugs could facilitate reducing the rising burden of ASCVD in China. Moderate-intensity statins, as the first-line medication for both primary and secondary prevention for ASCVD, should be prescribed in adequate doses for eligible people. Second, benefiting from the centralized medicine procurement policy in China, the annual cost of treatment per patient with moderate-intensity statins has reduced from $300 to $50 (18), making the treatment more affordable. The next step should be to ensure that statins is more widely available, given its unavailability in nearly half of the primary care institutions, especially rural village clinics (29). Third, there are still around 11 million Chinese people at high or very-high ASCVD risk requiring ezetimibe and/or PCSK9 inhibitors; however, the prices of the two drugs in China (i.e., annual $380 for ezetimibe, and $5,182 for evolucumab 140 mg per patient) are even higher than those in some high-income countries (25). Thus, it is necessary to promote the development of generic non-statin agents, and to reduce the price through pursuing centralized medicine procurement or relevant national policies.

Furthermore, it is worth noting that other lipid factors related to the development and progression of ASCVD and new effective LLT would indicate more choices and chances for the better management of dyslipidemia and ASCVD. On the one hand, guidelines have suggested that apolipoprotein B, Lp(a), and triglycerides may improve risk stratification (6), helping identify potential individuals at high risk. On the other hand, studies have reported that the lipid-lowering nutraceuticals (e.g., soluble fibers, plant sterols and stanols, etc.) could serve as a therapeutic tool to support lifestyle improvement in managing plasma lipid levels in low-risk population with mild-to-moderate dyslipidemia and help optimize LLT (30). Moreover, the emerging lipid-lowering medications, such as bempedoic acid, evinacumab, and inclisiran, have been proved effective in decreasing the LDL-C levels (31–33). These treatments could provide new options particularly for statin-intolerant subjects, and improve the adherence of LLT. Further studies are need to estimate the efficiency and the cost-effectiveness of these emerging agents in reducing ASCVD risk.

Findings in our study should be interpreted in the context of several limitations. First, our study used a convenient, rather than a random sampling design. However, the potential bias might be small, as the demographic profiles of population at the selected study sites were highly consistent with the national census data; and we did age- and sex-standardization based on the national population census of China to report the population-based estimation. Second, the concentration of LDL-C in our study was calculated using the Friedewald equation rather than directly measured, which might result in an underestimation of LDL-C levels in the setting of high triglyceride levels (34). Third, the estimation for treatment effect of lipid-lowering medications was based on the findings in clinical trials in which few Chinese participants were included. Nevertheless, previous studies had found that the pharmacokinetics and effectiveness of atorvastatin were similar between Western and Asian populations (35). Fourth, we assumed that participants in need of LLT in our study had received sufficient lifestyle modifications. However, the adherence to healthy lifestyle was usually poor in China (36). It should be emphasized that healthy lifestyle should be prioritized in the reduction of both LLT and ASCVD burden. Finally, for the sake of simplification, we simulated treatment scenarios without considering nonadherence to statins or intolerance, which occurred in 6.4% of Chinese population (37). Thus, the actual proportion requiring non-statin therapy might be slightly larger.

## Conclusions

In conclusion, in a simulation study based on the data from a nationwide population-based screening project, we found that one seventh of Chinese population aged 35–75 years were at high or very-high ASCVD risk; and one sixth required LLT. Moderate-intensity statin therapy was pivotal and relatively cost-effective for achieving optimal LDL-C goals; meanwhile around 10–25% of high- or very-high-risk patients would require additional non-statin therapy. Our study provides critical implications for the development of strategies for optimizing lipid management to reduce cardiovascular risk in China.

## Data Availability Statement

The original contributions presented in the study are included in the article/Supplementary Materials, further inquiries can be directed to the corresponding authors.

## Ethics Statement

The studies involving human participants were reviewed and approved by the Central Ethics Committee at the Fuwai Hospital. The patients/participants provided their written informed consent to participate in this study.

## Author Contributions

LB, XZhe, and XL designed the study. JY, LB, and XZha did statistical analysis. CW and SH verified the statistical results. LB drafted the manuscript, with further contributions from XZhe, XL, JLu, JLi, HZ, YG, YY, JC, WX, and LS. All authors interpreted the data and approved the final version of the article.

## Funding

This work was supported by the National Key Research and Development Program from the Ministry of Science and Technology of China [2018YFC1311205] and [2020YFC2004703]; the Chinese Academy of Medical Sciences Innovation Fund for Medical Science [2017-I2M-2-002], and the Major Public Health Service Project from the Ministry of Finance and National Health and Family Planning Commission of China.

## Conflict of Interest

The authors declare that the research was conducted in the absence of any commercial or financial relationships that could be construed as a potential conflict of interest.

## Publisher's Note

All claims expressed in this article are solely those of the authors and do not necessarily represent those of their affiliated organizations, or those of the publisher, the editors and the reviewers. Any product that may be evaluated in this article, or claim that may be made by its manufacturer, is not guaranteed or endorsed by the publisher.
