# Supplementary material for: Atherosclerotic Cardiovascular Disease Risk and Lipid-Lowering Therapy Requirement in China
Source: Front Cardiovasc Med. 2022 Mar 28;9:839571. doi: 10.3389/fcvm.2022.839571 (PMC8996051; doi:10.3389/fcvm.2022.839571)
Supplement: Supplementary file 1 [file Data_Sheet_1.pdf]

Supplementary Material

Lei Bi, Jiayi Yi, Chaoqun Wu, Shuang Hu, Xingyi Zhang, Jiapeng Lu, Jiamin Liu, Haibo Zhang, Yang Yang, Jianlan Cui, Wei Xu, Lijuan Song, Yuanlin Guo, Xi Li, Xin Zheng.
Atherosclerotic Cardiovascular Disease Risk and Lipid-lowering Therapy Requirement in China.

Supplementary Methods.
Supplementary Table 1. Characteristics of participants in the initial screening, those excluded, and the study population included in the analysis
Supplementary Table 2. Characteristics of participants assumed as not receiving lipid-lowering therapy and participants taking guideline-recommended lipid-lowering medications
Supplementary Table 3. Mean and SD percentage reduction in LDL-C with statins, ezetimibe, and PCSK9 inhibitors
Supplementary Table 4. Use of lipid-lowering therapy among participants failing to achieve the LDL-C goals
Supplementary Table 5. Estimated 95% CI for the proportion of participants by lipid-lowering therapy in the base-case scenario
Supplementary Table 6. Estimated 95% CI for the proportion of participants achieving LDL-C goals in the base-case scenario
Supplementary Table 7. Proportion of lipid-lowering therapy use in men and women before and after full treatment intensification in the base-case scenario
Supplementary Table 8. The proportion achieving LDL-C goals in men and women before and after full treatment intensification in the base-case scenario
Supplementary Table 9. Mean (SD) levels of LDL-C in men and women before and after full treatment intensification in the base-case scenario
Supplementary Table 10. Mean (SD) levels of LDL-C at each step in the base-case scenario and the final step in scenarios S1 to S7
Supplementary Table 11. Proportion of lipid-lowering therapy use after full treatment intensification across scenarios S1to S7
Supplementary Table 12. Estimated proportion of LDL-C goals achievement across scenarios S1 to S7
Supplementary Figure 1. Population pyramid charts of the age and sex compositions in the 2010 population census of China, the regular inhabitants aged 35–75 years at the selected 284 sites, and the participants in the current study
Supplementary Figure 2. Classification of low, moderate, high, and very-high risk for ASCVD according to the 2016 Chinese Guideline for the Management of Dyslipidemia in Adults
Supplementary Figure 3. Logic of lipid-lowering therapy simulation and proportion of very-high-risk patients flowing through the treatment intensification logic in the base-case scenario
Supplementary Figure 4. Use of lipid-lowering therapy and distribution of LDL-C levels among patients at high and very-high ASCVD risk after adding atorvastatin, and add-on ezetimibe in the base-case scenario

Supplementary Methods

Detailed information about sampling selection

From September 2015 to March 2020, we selected 284 sites (168 rural counties, 116 urban districts) from all the 31 provinces based on their geographic locations within each province, the number of residents living in rural or urban area, minority ethnicity distribution, quality of disease and death registries, and local capacity to support the project (see **Figure** below). Specifically, staff in the provincial coordinating office collected basic information (geographic information, economic development, population size, and minority ethnicity distribution) about the selected sites in their province; reported this information to the national coordinating office; and discussed with staff in the national coordinating office to determine the study sites. In each site, about 8-9 towns or sub-districts were chosen according to their population size, population stability (e.g., no sudden significant change in the number of residents), local staff's commitment, and ability to perform the screening. Initial screening stations were set up in each town or sub-district health center.

Potentially eligible participants were identified in each town or sub-district through official residential records and then invited by local community workers via telephone or extensive publicity campaigns on television and in newspaper. All participants were required to bring their identity cards to the screening clinics to verify that they met the inclusion criteria: 1) aged 35 to 75 years; 2) registered in the selected site's Hukou (a record officially identifying a person as a resident of an area), and had lived in the selected regions at least 6 months during the last 12 months. After the verification, eligible participants who had signed the informed consent agreement were then enrolled in the project (1).

Figure. Study sites in China PEACE Million Persons Project

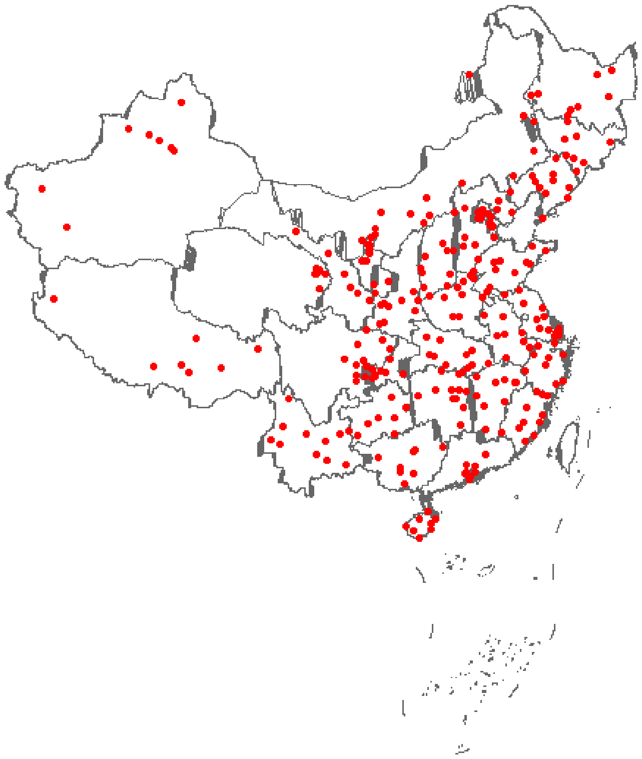

Supplementary Table 1. Characteristics of participants in the initial screening, those excluded, and the study population included in the analysis

Characteristics	Screened Participants	Excluded	Study population
N (%)	3 110 789 (100)	205 875 (6.6)	2 904 914 (93.4)
Age group, years			
35-44	464 485 (14.9)	33 378 (16.2)	431 107 (14.8)
45-54	964 740 (31.0)	64 517 (31.3)	900 223 (31.0)
55-64	976 544 (31.4)	62 873 (30.6)	913 671 (31.5)
65-75	705 020 (22.7)	45 107 (21.9)	659 913 (22.7)
Women	1 868 114 (60.0)	107 864 (52.4)	1 760 250 (60.6)
Urbanity			
Urban	1 240 659 (39.9)	80 084 (38.9)	1 160 575 (40.0)
Rural	1 870 130 (60.1)	125 791 (61.1)	1 744 339 (60.0)
Region			
Eastern	1 296 506 (41.7)	85 360 (41.5)	1 211 146 (41.7)
Central	725 048 (23.3)	46 485 (22.6)	678 563 (23.4)
Western	1 089 235 (35.0)	74 030 (35.9)	1 015 205 (34.9)
Household income, Yuan/year			
<10 000	578 463 (18.6)	37 806 (18.4)	540 657 (18.6)
10 000–50 000	1 693 842 (54.5)	112943 (54.9)	1 580 899 (54.4)
>50 000	538 927 (17.3)	36595 (17.8)	502 332 (17.3)
Unknown*	299 489 (9.6)	18463 (8.9)	281 026 (9.7)
Health insurance status			
Insured	3 041 407 (97.8)	202 266 (98.2)	2 839 141 (97.7)
Uninsured	19 018 (0.6)	1232 (0.6)	17 786 (0.6)
Unknown*	50 364 (1.6)	2377 (1.2)	47 987 (1.7)
Lipid-lowering therapy	87 917 (2.8)	12 480 (6.1)	75 437 (2.6)
Lipid levels, mmol/L			
Triglycerides (IQR)	1.33 (0.96)	1.23 (3.92)	1.33 (0.92)
Total cholesterol (SD)	4.56 (1.10)	4.60 (1.76)	4.56 (1.03)
HDL cholesterol (SD)	1.44 (0.42)	1.50 (0.64)	1.43 (0.40)
LDL cholesterol (SD)	2.42 (0.88)	2.29 (1.08)	2.42 (0.87)
Cardiovascular risk factors			
Hypertension	1 470 293 (47.4)	101 689 (49.4)	1 368 604 (47.1)
Diabetes mellitus	239 525 (7.7)	20 458 (9.9)	219 067 (7.5)
Current smoker	616 046 (19.8)	50 531 (24.5)	565 515 (19.5)
Obesity†	515 238 (16.6)	32 731 (15.9)	482 507 (16.6)

Data are N (%) if not otherwise indicated.

* Participants either refused to answer the question or did not know the answer.

† Defined as BMI ≥28 kg/m^2.

HDL, high-density lipoprotein; LDL, low-density lipoprotein; IQR, interquartile range; SD, standard deviation.

Definition of lipid-lowering therapy

Among the 75 437 participants with self-reported lipid-lowering medications, 35 352 (46.9%) recalled the name of the medications (generic or brand name), in whom 90.1% reported using guideline-recommended lipid-lowering medications (i.e., statin, Xuezhikang, or ezetimibe). 40 085 (53.1%) of the treated participants did not recall the name of their lipid-lowering medications. Xuezhikang is a partially purified extract of red yeast rice, which was reported could decrease the LDL-C levels and the recurrence of major coronary events in Chinese patients who experienced a previous myocardial infarction in the China Coronary Secondary Prevention Study (CCSPS) (2). And Xuezhikang 1.2 g is recommended as a moderate-intensity lipid-lowering therapy in the 2016 Chinese Guideline for the Management of Dyslipidemia in Adults (3).

In the simulation, we assumed those treated participants who were not taking guideline-recommended lipid-lowering medications or did not recall the name of their lipid-lowering medications as not receiving lipid-lowering therapy (LLT). The baseline characteristics of participants taking guideline-recommended lipid-lowering medications, and those assumed as not receiving LLT are shown in **Supplementary Table 2**. Comparing with the participants assumed as not receiving LLT, those taking guideline-recommended lipid-lowering medications tended to be older, more likely to be male, to live in urban area, have higher household income, be insured, be at very-high ASCVD risk, and have much lower lipid levels.

Supplementary Table 2. Characteristics of participants assumed as not receiving lipid-lowering therapy and participants taking guideline-recommended lipid-lowering medications

Characteristics	Assumed as not receiving LLT	Receiving guideline-recommended LLT	P Value
N (%)	43 587 (57.8)	31 850 (42.2)	
Age group, years			<0.0001
35-44	1808 (4.1)	726 (2.3)	
45-54	8715 (20.0)	5518 (17.3)	
55-64	17 650 (40.5)	13 180 (41.4)	
65-75	15 414 (35.4)	12 426 (39.0)	
Women	27 460 (63.0)	18 529 (58.2)	<0.0001
Urbanity			<0.0001
Urban	20 856 (47.9)	18 241 (57.3)	
Rural	22 731 (52.1)	13 609 (42.7)	
Region			<0.0001
Eastern	24 930 (57.2)	14 327 (45.0)	
Central	8743 (20.1)	9635 (30.2)	
Western	9914 (22.7)	7888 (24.8)	
Household income, Yuan/year			<0.0001
<10 000	7138 (16.4)	4720 (14.8)	
10 000–50 000	21 413 (49.1)	16 293 (51.2)	
>50 000	10 122 (23.2)	8783 (27.6)	
Unknown*	4914 (11.3)	2054 (6.4)	
Health insurance status			<0.0001
Insured	42 598 (97.7)	31 498 (98.9)	
Uninsured	245 (0.6)	75 (0.2)	
Unknown*	744 (1.7)	277 (0.9)	
Lipid levels, mmol/L			
Triglycerides (IQR)	1.63 (1.14)	1.48 (1.03)	<0.0001
Total cholesterol (SD)	4.65 (1.20)	4.10 (1.05)	<0.0001
HDL cholesterol (SD)	1.38 (0.39)	1.37 (0.37)	0.0585
LDL cholesterol (SD)	2.42 (1.02)	1.96 (0.88)	<0.0001
ASCVD risk			<0.0001
Low-risk	12 408 (28.5)	10 708 (33.6)	
Moderate-risk	8673 (19.9)	5431 (17.1)	
High-risk	15 713 (36.0)	6592 (20.7)	
Very-high-risk	6793 (15.6)	9119 (28.6)	
Cardiovascular risk factors			
Hypertension	34 695 (79.6)	25 871 (81.2)	<0.0001
Diabetes mellitus	16 112 (37.0)	8556 (26.9)	<0.0001

Current smoker	7796 (17.9)	5451 (17.1)	0.006
Obesity†	12 010 (27.6)	8682 (27.3)	0.370

Data are N (%) if not otherwise indicated.

* Participants either refused to answer the question or did not know the answer.

† Defined as BMI ≥28 kg/m^2.

ASCVD, atherosclerotic cardiovascular disease; HDL, high-density lipoprotein; LDL, low-density lipoprotein; LLT, lipid-lowering therapy; IQR, interquartile range; SD, standard deviation.

Supplementary Table 3. Mean and SD percentage reduction in LDL-C with statins, ezetimibe, and PCSK9 inhibitors

Drug	Dose, mg	Mean (Reference)	SD (Reference)
Atorvastatin	10	35.5%[4]	10.6%[5,6]
	20	41.4%[4]	13.5%[5,6]
	40	46.2%[4]	12.5%[5,6]
	80	50.2%[4]	13.8%[5,6]
Fluvastatin	20	17.0%[6]	8.0%[6]
	40	23.0%[6]	10.0%[6]
	80	26.0%[6]	9.0%[6]
Lovastatin	10	21.0%[7]	10.1%[5]
	20	24.0%[8]	11.0%[8]
	40	30.0%[8]	11.0%[8]
	60	34.5%[5]	11.7%[5]
Pravastatin	10	20.0%[6]	11.0%[6]
	20	24.0%[6]	11.0%[6]
	40	30.0%[6]	13.0%[6]
	80	33.0%[7]	11.2%[5]
Rosuvastatin	5	38.8%[4]	13.2%[5]
	10	44.1%[4]	12.5%[5,6]
	20	49.5%[4]	13.3%[5,6]
	40	54.7%[4]	12.9%[5,6]
Simvastatin	5	23.0%[7]	11.0%[5,6]
	10	27.4%[4]	13.7%[5,6]
	20	33.0%[4]	10.4%[5,6]
	40	38.9%[4]	14.0%[5,6]
	80	45.0%[4]	11.7%[5,6]
Ezetimibe	10	22.7%[9]	16.5%[10]
Evolocumab	140 (biweekly)	59.0%[5,11]	26.9%[5,12]
Alirocumab	75 (biweekly)	48.6%[5,13]	25.0%[5,13]

Mean and SD of the LDL-C reduction were presented by Cannon et al (5). Estimates were obtained from clinical trials, or estimated using data from clinical trials (5). The effect of evolocumab 140 mg biweekly was estimated using data from intention-to-treat analyses of the FOURIER trial (5,12). The effect of alirocumab 75 mg biweekly was presented by Allahyari A et al (5,13).

Supplementary Table 4. Use of lipid-lowering therapy among participants failing to achieve the LDL-C goals

ASCVD risk stratification	Lipid-lowering therapy	Proportion
Low-risk	No lipid-lowering therapy	98.9%
	Unknown name or not guideline-recommended medications	0.8%
	Statins or ezetimibe	0.3%
Moderate-risk	No lipid-lowering therapy	98.0%
	Unknown name or not guideline-recommended medications	1.5%
	Statins or ezetimibe	0.5%
High-risk	No lipid-lowering therapy	95.7%
	Unknown name or not guideline-recommended medications	3.4%
	Statins or ezetimibe	0.9%
Very-high risk	No lipid-lowering therapy	85.6%
	Unknown name or not guideline-recommended medications	7.5%
	Statins or ezetimibe	6.9%

Supplementary Table 5. Estimated 95% CI for the proportion of participants by lipid-lowering therapy in the base-case scenario. Numbers are shown in percent

		Low-risk			Moderate-risk			High-risk			Very-high-risk		
**Step in simulation**	**Lipid-lowering therapy**	Mean	Lower CI	Upper CI	Mean	Lower CI	Upper CI	Mean	Lower CI	Upper CI	Mean	Lower CI	Upper CI
Add/uptitrate to atorvastatin 20 mg	No lipid-lowering therapy	90.7	90.7	90.7	78.4	78.4	78.4	43.6	43.6	43.6	-	-	-
	Unknown name or not guideline-recommended medications	0.6	0.6	0.6	1.2	1.2	1.2	2.0	2.0	2.0	-	-	-
	LMIS only	0.3	0.3	0.3	0.5	0.5	0.5	0.6	0.6	0.6	2.6	2.6	2.6
	HMIS only	8.4	8.4	8.4	19.9	19.9	19.9	53.8	53.8	53.8	97.3	97.3	97.3
	Ezetimibe only	<0.01	<0.01	<0.01	<0.01	<0.01	<0.01	<0.01	<0.01	<0.01	0.03	0.03	0.03
	HMIS + ezetimibe	-	-	-	-	-	-	<0.01	<0.01	<0.01	0.03	0.03	0.03
Add ezetimibe	No lipid-lowering therapy	-	-	-	-	-	-	43.6	43.6	43.6	-	-	-
	Unknown name or not guideline-recommended medications	-	-	-	-	-	-	2.0	2.0	2.0	-	-	-
	LMIS only	-	-	-	-	-	-	0.6	0.6	0.6	2.6	2.6	2.6
	HMIS only	-	-	-	-	-	-	42.5	42.5	42.5	72.9	72.9	72.9
	Ezetimibe only	-	-	-	-	-	-	<0.01	<0.01	<0.01	0.03	0.03	0.03
	HMIS + ezetimibe	-	-	-	-	-	-	11.3	11.3	11.3	24.5	24.5	24.5
Add evolocumab	No lipid-lowering therapy	-	-	-	-	-	-	43.6	43.6	43.6	-	-	-
	Unknown name or not guideline-recommended medications	-	-	-	-	-	-	2.0	2.0	2.0	-	-	-
	LMIS only	-	-	-	-	-	-	0.6	0.6	0.6	2.6	2.6	2.6
	HMIS only	-	-	-	-	-	-	42.5	42.5	42.5	72.9	72.9	72.9
	Ezetimibe only	-	-	-	-	-	-	<0.01	<0.01	<0.01	0.03	0.03	0.03
	HMIS + ezetimibe	-	-	-	-	-	-	6.5	6.5	6.5	13.2	13.2	13.2
	HMIS + ezetimibe + evolocumab	-	-	-	-	-	-	4.8	4.8	4.8	11.3	11.3	11.3

CI, confidence interval; HMIS, maximized uptake of moderate-intensity statins; LMIS, statins with doses less than HMIS.

Supplementary Table 6. Estimated 95% CI for the proportion of participants achieving LDL-C goals in the base-case scenario

Numbers are shown in percent

		Maximized uptake of MIS			Add ezetimibe			Add evolocumab		
**ASCVD risk**	**LDL-C goals**	Mean	Lower CI	Upper CI	Mean	Lower CI	Upper CI	Mean	Lower CI	Upper CI
Low-risk	LDL-C <3.4 mmol/L	99.8	99.8	99.8	-	-	-	-	-	-
Moderate-risk	LDL-C <3.4 mmol/L	99.6	99.6	99.6	-	-	-	-	-	-
High-risk	LDL-C <2.6 mmol/L	88.7	88.7	88.7	95.2	95.2	95.2	99.6	99.6	99.6
Very-high risk	LDL-C <1.8 mmol/L	75.5	75.5	75.5	88.7	88.7	88.7	99.0	99.0	99.0

MIS, moderate-intensity statin; CI, confidence interval.

Supplementary Table 7. Proportion of lipid-lowering therapy use in men and women before and after full treatment intensification in the base-case scenario

Numbers are shown in percent

		Low-risk		Moderate-risk		High-risk		Very-high-risk	
	**Lipid-lowering therapy**	**Before**	**After**	**Before**	**After**	**Before**	**After**	**Before**	**After**
**Men**	No lipid-lowering therapy	98.8	95.0	98.3	82.2	96.2	49.0	78.6	-
	Unknown name or not guideline-recommended	0.6	0.6	1.0	0.9	2.7	1.5	8.4	-
	Statin only	0.6	4.4	0.7	16.9	1.1	42.8	12.9	79.6
	Statin + ezetimibe	-	-	-	-	-	4.2	0.1	11.4
	Statin + ezetimibe + evolocumab	-	-	-	-	-	2.5	-	9.0
**Women**	No lipid-lowering therapy	98.7	88.5	97.2	75.7	92.4	36.3	82.7	-
	Unknown name or not guideline-recommended	0.7	0.6	1.7	1.3	5.4	2.7	8.1	-
	Statin only	0.6	10.9	1.1	23.0	2.2	43.7	9.2	71.4
	Statin + ezetimibe	-	-	-	-	-	9.6	-	15.0
	Statin + ezetimibe + evolocumab	-	-	-	-	-	7.7	-	13.6

Supplementary Table 8. The proportion achieving LDL-C goals in men and women before and after full treatment intensification in the base-case scenario

Numbers are shown in percent

ASCVD risk	LDL-C goals	Men		Women	
		Before	After	Before	After
Low-risk	LDL-C <3.4 mmol/L	96.2	99.9	89.7	99.8
Moderate-risk	LDL-C <3.4 mmol/L	83.7	99.6	78.0	99.4
High-risk	LDL-C <2.6 mmol/L	51.4	99.8	40.5	99.3
Very-high risk	LDL-C <1.8 mmol/L	37.2	99.1	26.5	98.6

Supplementary Table 9. Mean (SD) levels of LDL-C in men and women before and after full treatment intensification in the base-case scenario

Numbers are shown as Mean (SD), mmol/L

**ASCVD risk**	**Men**		**Women**	
	**Before**	**After**	**Before**	**After**
Low-risk	2.06 (0.72)	2.0 (0.63)	2.33 (0.80)	2.16 (0.62)
Moderate-risk	2.55 (0.81)	2.29 (0.57)	2.83 (0.73)	2.48 (0.48)
High-risk	2.67 (0.90)	1.90 (0.35)	3.08 (1.26)	1.87 (0.35)
Very-high risk	2.16 (0.86)	1.13 (0.16)	2.45 (0.96)	1.17 (0.16)

Supplementary Table 10. Mean (SD) levels of LDL-C at each step in the base-case scenario and the final step in scenarios S1 to S7

Scenario	ASCVD risk	Logic step	Mean (SD) LDL-C, mmol/L
Base-case Scenario	Low-risk	Add/uptitrate to atorvastatin 20 mg	2.11(0.62)
	Moderate-risk	Add/uptitrate to atorvastatin 20 mg	2.40 (0.53)
	High-risk	Add/uptitrate to atorvastatin 20 mg	2.06 (0.51)
		Add ezetimibe	1.97 (0.37)
		Add evolocumab 140 mg	1.89 (0.35)
	Very-high-risk	Add/uptitrate to atorvastatin 20 mg	1.43 (0.57)
		Add ezetimibe	1.30 (0.32)
		Add evolocumab 140 mg	1.15 (0.16)
Scenario S1	Low-risk*	No simulation	2.24 (0.78)
	Moderate-risk	Add/uptitrate to atorvastatin 20 mg	2.63 (0.69)
Scenario S2	High-risk	Add evolocumab 140 mg (removing ezetimibe)	1.91 (0.32)
	Very-high-risk	Add evolocumab 140 mg (removing ezetimibe)	1.19 (0.14)
Scenario S3	Very-high-risk	Add evolocumab 140 mg	1.16 (0.44)
Scenario S4	Very-high-risk	Add evolocumab 140 mg	1.13 (0.44)
Scenario S5	High-risk	Add evolocumab 140 mg	1.91 (0.32)
	Very-high-risk	Add evolocumab 140 mg	1.18 (0.28)
Scenario S6	High-risk	Add alirocumab 75mg	1.90 (0.33)
	Very-high-risk	Add alirocumab 75mg	1.17 (0.14)
Scenario S7	Very-high-risk	Add evolocumab 140 mg	1.00 (0.40)

*The proportion of people at low ASCVD risk with an LDL-C level of ≥4.1mmol/L was only 1.7%. Thus, we did not conduct LLT simulation in the low-risk participants in scenario S1.

Supplementary Table 11. Proportion of lipid-lowering therapy use after full treatment intensification across scenarios S1 to S7

Lipid-lowering therapy	Scenarios, % of participants										
	Scenario S1		Scenario S2		Scenario S3	Scenario S4	Scenario S5		Scenario S6		Scenario S7
	Low-risk	Moderate-risk	High-risk	Very-high-risk			High-risk	Very-high-risk	High-risk	Very-high-risk	
No lipid-lowering therapy	98.7	93.3	43.6	-	-	-	43.6	-	43.6	-	-
Unknown name or not guideline-recommended	0.7	1.4	2.0	-	-	-	2.0	-	2.0	-	-
Statin only	0.6	5.3	43.1	75.5	75.5	70.1	49.4*	88.5†	43.1	75.5	54.8
Statin + ezetimibe	-	-	6.5	13.2	14.6	17.4	1.6	3.5	6.5	13.2	25.6
Statin + evolocumab	-	-	4.8	11.3	-	-	-	-	-	-	-
Statin + ezetimibe + evolocumab	-	-	-	-	9.9	12.5	3.4	8.0	-	-	19.6
Statin + ezetimibe + alirocumab	-	-	-	-	-	-	-		4.8	11.3	-

*Including 43.1% with moderate-intensity statin monotherapy; 6.3% with high-intensity statin monotherapy (atorvastatin 40-80 mg).
†Including 75.5% with moderate-intensity statin monotherapy; 13.0% with high-intensity statin monotherapy (atorvastatin 40-80 mg).

Supplementary Table 12. Estimated proportion of LDL-C goals achievement across scenarios S1 to S7

Scenario	ASCVD risk	% in the study population
Scenario S1	Low-risk	98.3
	Moderate-risk	99.6
Scenario S2	High-risk	99.0
	Very-high-risk	97.1
Scenario S3	Very-high-risk	97.6
Scenario S4	Very-high-risk	99.0
Scenario S5	High-risk	99.5
	Very-high-risk	98.8
Scenario S6	High-risk	99.4
	Very-high-risk	98.3
Scenario S7	Very-high-risk	91.2

Supplementary Figure 1. Population pyramid charts of the age and sex compositions in the 2010 population census of China, the regular inhabitants aged 35–75 years at the selected 284 sites, and the participants in the current study

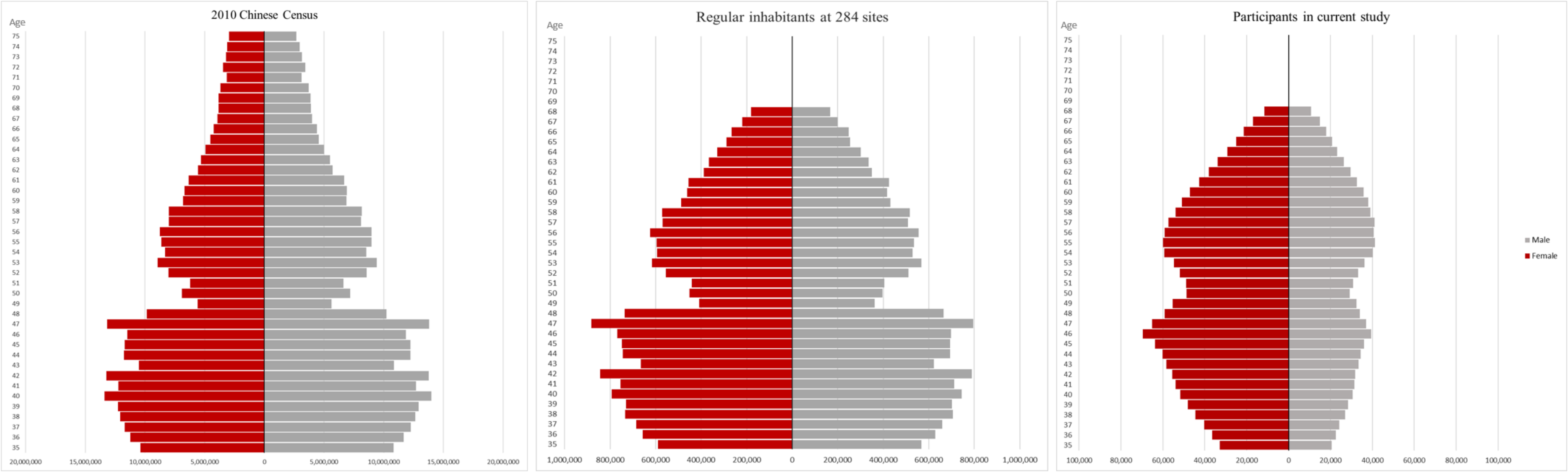

Our screening was from 2015 to 2020, so we adjust the age by minus 7 years in the regular inhabitants at the selected 284 sites and the participants in current study, to make it comparable with the data in the 2010 Chinese census.

Supplementary Figure 2. Classification of low, moderate, high, and very-high risk for ASCVD according to the 2016 Chinese Guideline for the Management of Dyslipidemia in Adults

Those who meet any of the following conditions can be directly classified as high-risk or very-high-risk groups

Very-high risk: patients with established ASCVD

High risk: (1) LDL-C ≥4.9 mmol/L or TC ≥7.2 mmol/L

(2) patients with diabetes [LDL-C 1.8–4.9 mmol/L (or TC 3.1–7.2 mmol/L) and age ≥40 years]

↓ Conditions not meet, evaluate the 10-year risk for ASCVD

Number of risk factors**		Classification of serum cholesterol levels (mmol/L)		
		3.1≤ TC <4.1 or 1.8≤ LDL-C <2.6	4.1≤ TC <5.2 or 2.6≤ LDL-C <3.4	5.2≤ TC <7.2 or 3.4≤ LDL-C <4.9
Without hypertension	0-1	Low risk (<5%)	Low risk (<5%)	Low risk (<5%)
	2	Low risk (<5%)	Low risk (<5%)	Moderate risk (5%–9%)
	3	Low risk (<5%)	Moderate risk (5%–9%)	Moderate risk (5%–9%)
With hypertension	0	Low risk (<5%)	Low risk (<5%)	Low risk (<5%)
	1	Low risk (<5%)	Moderate risk (5%–9%)	Moderate risk (5%–9%)
	2	Moderate risk (5%–9%)	High risk (≥10%)	High risk (≥10%)
	3	High risk (≥10%)	High risk (≥10%)	High risk (≥10%)

↓ For people with moderate 10-year risk for ASCVD and age <55, evaluate the lifetime risk for ASCVD

Those who meet any two or more of the following conditions can be directly classified as high-lifetime risk group

High risk: (1) Systolic blood pressure ≥160 mmHg or diastolic blood pressure ≥100 mmHg

(2) Non-HDL-C ≥5.2 mmol/L (200 mg/dl)

(3) HDL-C <1.0 mmol/L (40 mg/dl)

(4) BMI ≥28 kg/m^2

(5) Current smoking

**Risk factors include current smoking, low HDL-C level, and men with age ≥45 or women with age ≥55. ASCVD, atherosclerotic cardiovascular disease; BMI, body mass index; HDL-C, high-density lipoprotein cholesterol; LDL-C, low-density lipoprotein cholesterol; TC, total cholesterol.

Supplementary Figure 3. Logic of lipid-lowering therapy simulation and proportion of very-high-risk patients flowing through the treatment intensification logic in the base-case scenario

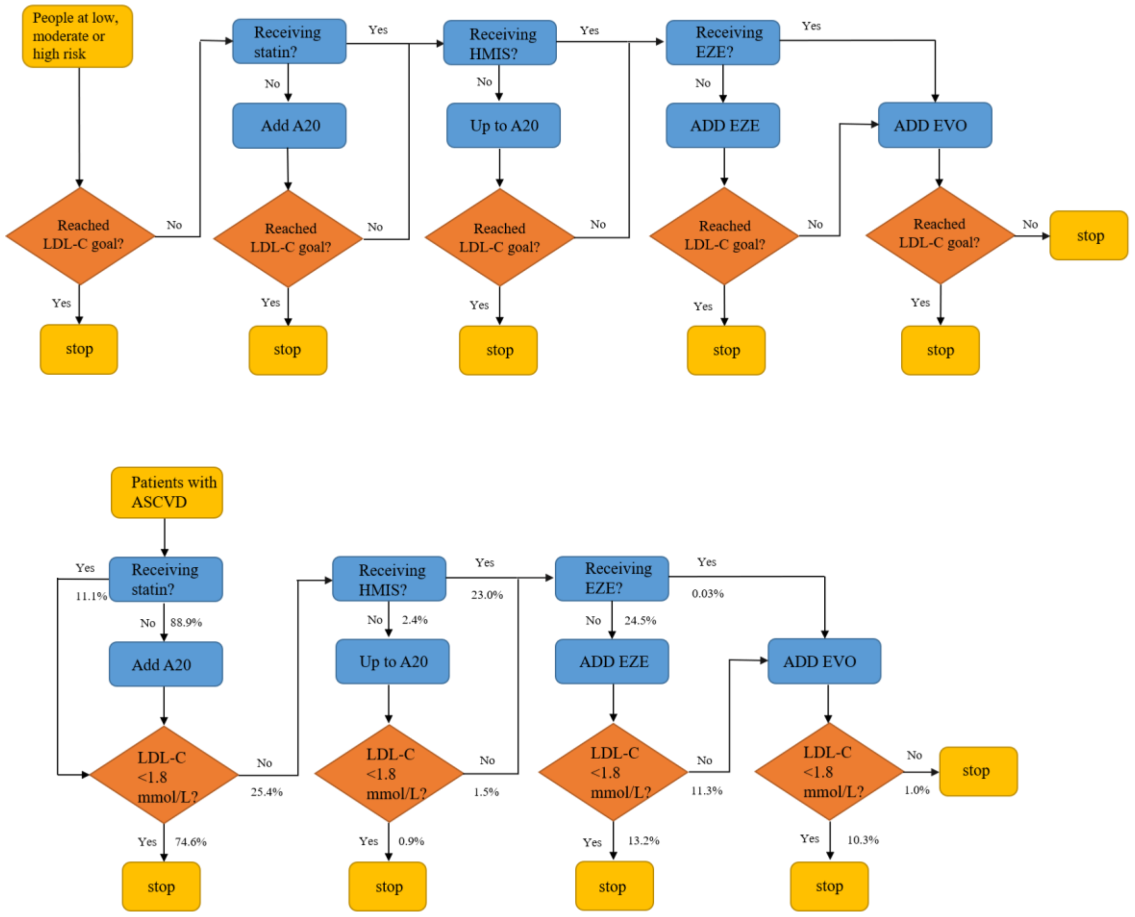

The goal level of LDL-C is <1.8 mmol/L for very-high-risk patients, <2.6 mmol/L for high-risk patients, and <3.4 mmol/L for low- or moderate-risk individuals, respectively. A20, atorvastatin 20 mg; HMIS, maximized uptake of moderate-intensity statins, including: atorvastatin 20 mg, simvastatin 40 mg, rosuvastatin 10 mg, pravastatin 40 mg, pitavastatin 4 mg, lovastatin 40 mg, or fluvastatin 80 mg; EZE, ezetimibe; EVO, evolocumab 140 mg, biweekly.

Supplementary Figure 4. Use of lipid-lowering therapy and distribution of LDL-C levels among patients at high and very-high ASCVD risk after adding atorvastatin, and add-on ezetimibe in the base-case scenario

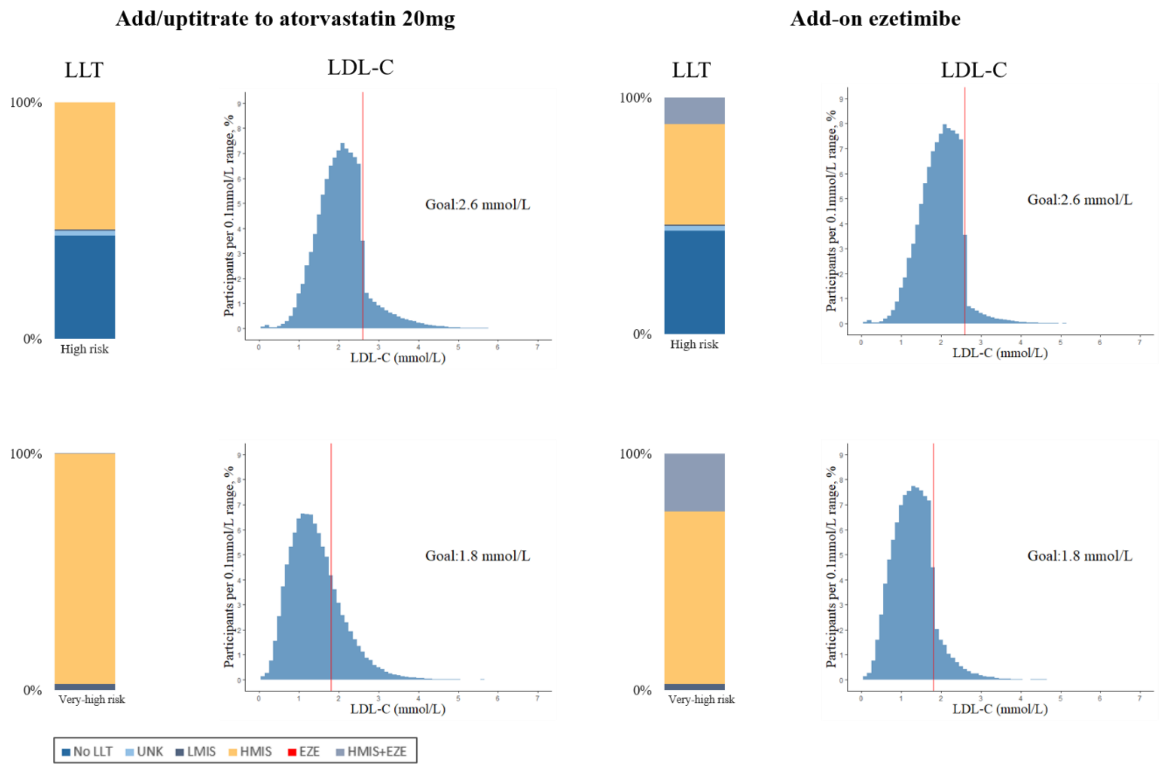

LLT, lipid-lowering therapy; UNK, unknown name or not guideline-recommended medications; LMIS, statins with dose less than HMIS; HMIS, maximized uptake of moderate-intensity statins; EZE, ezetimibe.

REFERENCES

1. Lu J, Xuan S, Downing NS, et al. Protocol for the China PEACE (Patient-centered Evaluative Assessment of Cardiac Events) Million Persons Project pilot. *BMJ Open*. (2016) 6(1):e010200. doi: 10.1136/bmjopen-2015-010200
2. Lu Z, Kou W, Du B, et al. Effect of Xuezhikang, an extract from red yeast Chinese rice, on coronary events in a Chinese population with previous myocardial infarction. *Am J Cardiol*. (2008)101(12):1689-93. doi: 10.1016/j.amjcard.2008.02.056
3. Joint committee issued Chinese guideline for the management of dyslipidemia in adults. 2016 Chinese guideline for the management of dyslipidemia in adults. *Zhonghua Xin Xue Guan Bing Za Zhi*. (2016) 44(10):833-53. doi: 10.3760/cma.j.issn.0253-3758.2016.10.005
4. Nicholls SJ, Brandrup-Wognsen G, Palmer M, Barter PJ. Meta-analysis of comparative efficacy of increasing dose of Atorvastatin versus Rosuvastatin versus Simvastatin on lowering levels of atherogenic lipids (from VOYAGER). *Am J Cardiol*. (2010) 105(1):69-76. doi: 10.1016/j.amjcard.2009.08.651
5. Cannon CP, Khan I, Klimchak AC, Reynolds MR, Sanchez RJ, Sasiela WJ. Simulation of Lipid-Lowering Therapy Intensification in a Population With Atherosclerotic Cardiovascular Disease. *JAMA Cardiol*. (2017) 2(9):959-966. doi: 10.1001/jamacardio.2017.2289
6. Ward S, Lloyd Jones M, Pandor A, et al. A systematic review and economic evaluation of statins for the prevention of coronary events. *Health Technol Assess*. (2007) 11(14):1-160, iii-iv. doi: 10.3310/hta11140
7. Law MR, Wald NJ, Rudnicka AR. Quantifying effect of statins on low density lipoprotein cholesterol, ischaemic heart disease, and stroke: systematic review and meta-analysis. *BMJ*. (2003) 326(7404):1423. doi: 10.1136/bmj.326.7404.1423
8. Bradford RH, Shear CL, Chremos AN, et al. Expanded Clinical Evaluation of Lovastatin (EXCEL) study results. I. Efficacy in modifying plasma lipoproteins and adverse event profile in 8245 patients with moderate hypercholesterolemia. *Arch Intern Med*. (1991) 151(1):43-9. doi: 10.1001/archinte.151.1.43
9. Cannon CP, Blazing MA, Giugliano RP, et al. Ezetimibe Added to Statin Therapy after Acute Coronary Syndromes. *N Engl J Med*. (2015) 372(25):2387-97. doi: 10.1056/NEJMoa141048
10. Descamps O, Tomassini JE, Lin J, et al. Variability of the LDL-C lowering response to ezetimibe and ezetimibe + statin therapy in hypercholesterolemic patients. *Atherosclerosis*. (2015) 240(2):482-9. doi: 10.1016/j.atherosclerosis.2015.03.004
11. Qamar A, Giugliano RP, Keech AC, et al. Interindividual Variation in Low-Density Lipoprotein Cholesterol Level Reduction With Evolocumab: An Analysis of FOURIER Trial Data. *JAMA Cardiol*. (2019) 4(1):59-63. doi: 10.1001/jamacardio.2018.4178
12. Sabatine MS, Giugliano RP, Keech AC, et al. Evolocumab and Clinical Outcomes in Patients with Cardiovascular Disease. *N Engl J Med*. (2017) 376(18):1713-1722. doi: 10.1056/NEJMoa1615664
13. Allahyari A, Jernberg T, Hagstrom E, Leosdottir M, Lundman P, Ueda P. Application of the 2019 ESC/EAS dyslipidaemia guidelines to nationwide data of patients with a recent myocardial infarction: a simulation study. *Eur Heart J*. (2020) 41:3900-3909. doi: 10.1093/eurheartj/ehaa034
